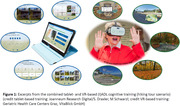# A tablet‐ and virtual reality‐based training for preventing and detecting cognitive decline: a usability study

**DOI:** 10.1002/alz.094939

**Published:** 2025-01-09

**Authors:** Julia Zuschnegg, Anna Schultz, Lucas Paletta, Amir Dini, Amadeus Linzer, Ursula Berger, Wolfgang Kratky, Judith Goldgruber, Marisa Koini, Silvia Russegger, Sandra Draxler, Thomas Orgel, Michael Schneeberger, Martin Pszeida, Wolfgang Weiss, Sandra Schüssler

**Affiliations:** ^1^ Medical University of Graz, Graz, Styria Austria; ^2^ JOANNEUM RESEARCH Forschungsgesellschaft mbH, Graz, Styria Austria; ^3^ VitaBlick GmbH, Oberwart, Burgenland Austria; ^4^ Geriatric Health Care Centers Graz, Graz, Styria Austria

## Abstract

**Background:**

Virtual Reality (VR) is hailed as a top emerging technology for older adults in healthcare. Despite its potential, limited research exists on VR applications for individuals with cognitive decline, particularly in leisure‐based cognitive training, as opposed to traditional (instrumental) activities of daily living (I)ADL training such as shopping. The SmartAktiv project has developed immersive VR leisure experiences with (I)ADL cognitive training embedded, aiming to enhance cognition in a playful manner and detect early cognitive deficits. We assessed the usability of the SmartAktiv VR‐based intervention combined with a tablet‐based training among participants before initiating a pilot study.

**Method:**

Healthy older adults (n = 4), people with suspected mild cognitive impairment (MCI) (n = 4), people with suspected dementia (n = 4) (according Montreal Cognitive Assessment, MoCA) and healthcare professionals (n = 4) tested the combined tablet‐ and VR‐based training (hiking tour scenario). After the intervention, qualitative focus groups were held with each group to gather insights into training’s usability from participants' experiences. Furthermore, cybersickness (Simulator Sickness Questionnaire, SSQ) was measured.

**Result:**

Older adults (MoCA, x̅ 27.00±1.41 points), people with suspected MCI (MoCA, x̅ 23.25±1.26 points) those with suspected dementia (MoCA, x̅ 16.00±0.00 points) and healthcare professionals experienced negligible cybersickness after the training (SSQ, x̅ 6.98±5.26 points). Qualitative findings indicated positive perceptions of the intervention among all participants. Participants appreciated the variety of cognitive exercises (e.g., quiz, puzzle) in the tablet‐training program, but emphasized the need for greater sensitivity of the tablet‐PC and the necessity of a tablet pen. The immersive VR hiking tour was very well‐received, evoking a longing for nature, especially among older individuals (with/without cognitive impairment). They found the interactive cognitive (I)ADL tasks enjoyable/fun, with some even reporting feeling more alert/euphoric afterwards. However, hand tracking issues arose in certain VR tasks (e.g., picking mushrooms), and low contrast made some VR elements difficult to perceive (e.g., payment for train).

**Conclusion:**

The findings offer insights into the usability of the combined tablet‐ and VR‐based (I)ADL cognitive training, to be considered for a pilot study. This pilot study will test four scenarios (hiking tour, beach vacation, city trip, winter outing) on individuals (n = 30) with and without cognitive decline.